# Utilization and determinants of adequate quality antenatal care services in India: evidence from the National Family Health Survey (NFHS-5) (2019-21)

**DOI:** 10.1186/s12884-023-06117-z

**Published:** 2023-11-17

**Authors:** Siaa Girotra, Mansi Malik, Shubhanjali Roy, Saurav Basu

**Affiliations:** Indian Institute of Public Health, Public Health Foundation of India, New Delhi, India

**Keywords:** Antenatal Care, Pregnancy and Childbirth, ANC Quality, NFHS

## Abstract

**Background:**

Pregnancy-related complications and insufficiencies in antenatal care services are leading causes of maternal and infant morbidity and mortality in low-resource settings. However, there has been an undue focus on achieving a minimum number of Antenatal Care (ANC) visits without adequate focus on the factors affecting ANC service utilization. This secondary data analysis from the fifth round of the National Family Health Survey (NFHS-5, 2019-21) was conducted to estimate the coverage of adequate quality ANC service and its determinants in India.

**Methods:**

The study sample included 176,877 women aged 15–49 years who had experienced a pregnancy in the last 5 years. The primary outcome variable was the utilization of ANC services by women during their last pregnancy assessed by the frequency of ANC visits and the quality of ANC services. Quality of ANC service utilisation was categorised as adequate quality, inadequate quality and ≥ 4 ANC visits and, inadequate quality and < 4 ANC visits. We performed multinomial logistic regression and reported relative risk ratio (RRR) along with 95% confidence intervals. We adjusted for sampling weight, clustering, and stratification in the sampling design.

**Results:**

The median (IQR) number of ANC visits attended by a woman during her previous pregnancy was 4 (IQR 3–7). A majority (59.25%) of the women reported availing of ≥ 4 antenatal care (ANC) visits during their previous pregnancy while 6.12% of women reported availing no ANC visits in their last pregnancy. Women aged ≥ 30 years were significantly less likely (aRRR 0.73 95% CI 0.66, 0.80) to receive ANC services of inadequate quality, and < 4 ANC visits. Additionally, any exposure to mass media (aRRR 0.69 95% CI 0.66, 0.73), and having health insurance (aRRR 0.71 95% CI 0.68, 0.75) decreased their risk of receiving inadequate quality ANC services and < 4 ANC visits. Women belonging to the richest wealth quintile (aRRR 0.52 95% CI 0.47,0.58) and those with an intended pregnancy (aRRR 0.62 95% CI 0.58 ,0.66) were at significantly lower risk of utilizing inadequate quality ANC services and < 4 ANC visits.

**Conclusion:**

Although nearly 3 in 5 women in India utilized a minimum mandated ≥ 4 ANC visits during their last pregnancy, only one in five of those received adequate quality of ANC services indicating suboptimal content. However, only one in five women utilized the WHO-mandated ≥ 8 ANC visits for a positive pregnancy experience. Furthermore, 14.3% of the women received ANC services of inadequate quality despite attending ≥ 4 ANC visits in their previous pregnancy. Our study emphasized the importance of the quality of ANC services utilised irrespective of number of ANC visits availed. Efforts should be undertaken to enhance the utilization of antenatal care (ANC) services by implementing media initiatives that aim to raise awareness, particularly among women belonging to disadvantaged population groups.

**Supplementary Information:**

The online version contains supplementary material available at 10.1186/s12884-023-06117-z.

## Introduction

Pregnancies are a physiological state characterized by the risk of adverse events and complications that can adversely impact the mother and her fetus [1]. A high maternal and infant mortality burden is prevalent in the developing world with 94% of these deaths restricted to lower-middle-income countries (LMICs) [2–4].

Antenatal care (ANC) is a component of the continuum of reproductive health care to monitor and safeguard the well-being of the mother and fetus through early detection of high-risk pregnancies and complications through screening, with improved access to care, health education, vaccination, and medical therapy as required that promote positive health outcomes for both the mother and her fetus [1, 5].

The WHO recommends a minimum of four sufficiently high quality ANC visits to achieve optimal health outcomes that should be completed before delivery [1]. The inadequacy of ANC visits is linked to adverse health outcomes including an elevated risk of LBW, preterm delivery, abortion, and perinatal mortality [6, 7]. However, globally, across LMICs, the reduction in maternal and infant mortality does not correspond to the significant increase in ANC coverage, indicating a high burden of suboptimal ANC quality driving persistently adverse maternal and newborn health outcomes [8].

In India, the country with the highest maternal and birth cohort in the world, the current estimated maternal mortality rate (MMR) of 7.2 and infant mortality rate (IMR) of 30 per 1,000 live births despite substantial reduction is not on track to meet sustainable development goal (SDG) targets by 2030 [9]. Full ANC coverage defined as “≥4 ANC visits, at least one tetanus toxoid (TT) injection, and consumption of iron folic acid (IFA) for a minimum of 100 days during pregnancy” has increased from 27% to 46% between 1992 and 2016, which can be attributed to the introduction and effective implementation of multiple national health programs [10–13]. Nevertheless, key quality metrics for ANC care indicate suboptimal quality parameters, especially in public healthcare settings that provide free-of-cost ANC services, especially to the socioeconomically vulnerable population. As per a large nationally representative cross-sectional survey NFHS-4 (2015-16) during their last pregnancy, 89.41% of women reported getting their blood pressure measured while 87.94% of women had a urine test, 87.30% of women had a blood test, and 83.51% women were administered tetanus vaccines (at least 2 doses). Only 18.19% reported being administered deworming medication while 77.97% of women reported consuming iron tablets/syrups during their last pregnancy [13].

India is committed to achieving the SDG target of reducing the maternal mortality ratio to less than 70 per 100,000 live births and ending malnutrition (SDG Target 3.1 and 2.2) which requires enhanced ANC services reaching the unreached pregnant mothers [14]. The current WHO-positive pregnancy guidelines outline five areas to enhance the use and effectiveness of ANC services, one of which is to raise the advised number of ANC visits from at least four to at least eight contacts throughout the course of pregnancy [1].

**Fig. 1:**
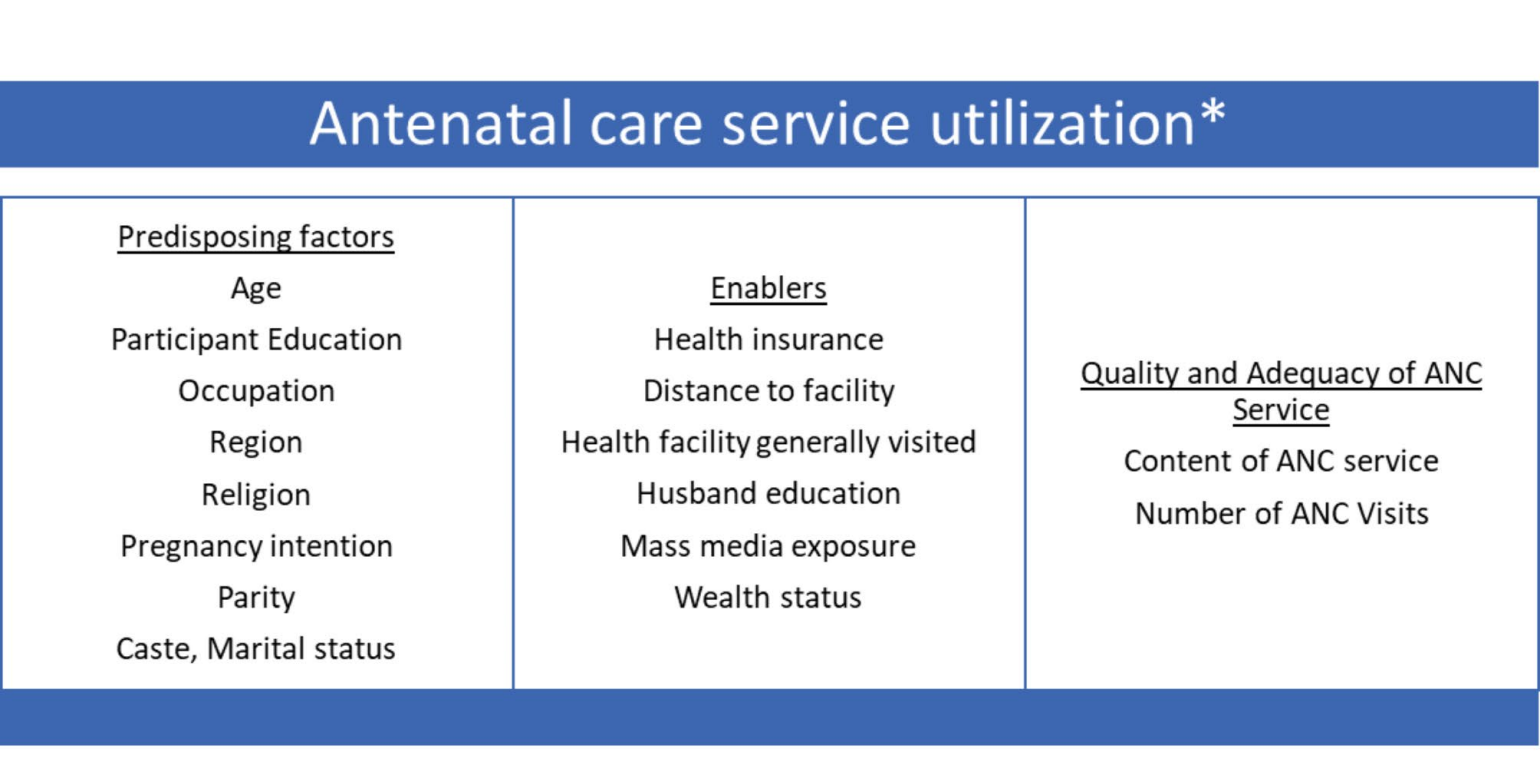
Conceptual Framework for ANC Service Utilization. *Based on Andersen and Newman Framework of Health Services Utilization

ANC service access and utilization can be influenced by individual, community, and health-system determinants. The Andersen and Newman Framework of Health Services in this context explains ANC utilization by three broad components (Fig. 1): Predisposing factors including sociodemographic determinants such as education and socioeconomic status, enabling factors including factors such as the availability of health insurance and accessibility of health facilities, while quality and adequacy of ANC services can be impacted by the expertise of ANC providers, staff motivation, budgetary provisions, integration with other health programs, and accessibility to consumables, drugs, and basic equipment [15, 16]. For instance, in the Indian context, frontline health workers known as Accredited Social Health Activists (ASHAs) are entrusted with improving accessibility to health systems by linkage of pregnant mothers (clients) and the public (primary) health system which provides universal free of cost essential antenatal care services [17].

According to a study conducted in Nepal, women who received care from qualified professionals had more effective ANC including at least four visits received health instructions, iron supplements, tetanus toxoid injections, and blood pressure screening [18]. However, there is a lack of current epidemiological evidence from large survey data from India ascertaining the ANC service coverage with its quality assessment and recognition of their sociodemographic, clinical, and health-system determinants.

We, therefore, conducted a secondary data analysis from the current round of the National Family Health Survey (NFHS-5, 2019-21) with the objective of estimating the coverage of adequate quality ANC service and its determinants in India. The findings of this study would contribute to strengthening the reproductive and child health program of India at both the country and the state levels.

## Methods

### Data source

Our analysis makes use of individual-level data from the National Family Health Survey 5 (NFHS-5) of India, which was carried out between 2019 and 21. The National Family Health Surveys (NFHS) is a set of cross-sectional, nationally representative surveys that collect information on a variety of demographic, socioeconomic, maternal, and child health outcomes, reproductive health, and family planning aspects. Using a two-stage stratified sampling strategy, approximately 724,115 women between the ages of 15 and 49 from 636,699 households were questioned in the fifth round, with a 92% response rate. Information on the sampling strategy and the tools utilized is accessible elsewhere [19]. Women’s individual recode file (IAIR7AFL) was utilized for the analysis. The study sample was 176,877 which consisted of women aged 15–49 with a pregnancy who had their last child in the past 5 years preceding the survey.

### Operational definitions

#### Outcome variable

The utilization of ANC services by women belonging to the reproductive age-group during their last pregnancy was assessed in terms of the frequency of ANC visits and the quality of ANC services utilized.

1. Adequate Quality.
2. Inadequate quality and ≥ 4 ANC visits.
3. Inadequate quality and < 4 ANC visits.

##### Quality of ANC

As per WHO guidelines [1], a core set of ANC services includes blood pressure measurement, a urine test, a blood test, tetanus vaccines (at least 2 doses), and administration of deworming, Iron-folic acid, and anti-malaria medication during the pregnancy. Information on all of these factors was available in the survey, except for anti-malarial medication. Moreover, we excluded deworming medication as it is not a mandatory service in all districts of India. Therefore, a composite quality score was generated for the rest of the factors. Every ‘Yes’ response was coded as + 1 and the ‘no’ or missing response was assigned 0 with the total score ranging from 0–5. Based on a previous study by Dickson et al (2023), we operationally defined a score of 5 as utilization of an adequate quality of ANC services and a score below 5 considered as an inadequate quality of ANC services [20].

### Independent variables

Individual level factors such as age, marital status, education level of mother and father, occupation, and other lifestyle factors such as mass media exposure, and tobacco or alcohol consumption were included. Household characteristics consisted of religion, caste, health insurance type, and wealth index. Additionally, other pregnancy characteristics such as parity, the intention of pregnancy, ANC provider, and distance to health facility were included.

Detailed description of other variables can be found in Additional File 1.

### Statistical analysis

The analysis was restricted to the antenatal care utilized during the last pregnancy of women aged 15–49 with a pregnancy who had their last child in the past 5 years preceding the survey. Women who availed no ANC services were excluded from the analysis. Independent variables were presented descriptively along with weighted percentages stratified by the adequacy of the quality of ANC service utilization.

Multinomial logistic regression was used to assess the determinants of the quality of ANC service utilization among women. In the model, high-quality ANC service utilization was considered as the reference and the adjusted Relative Risk Ratio (aRRR) along with 95% confidence intervals were reported. Based on epidemiological and statistical significance, variables were added to the multivariable model. A p-value of < 0.05 was considered statistically significant for all analyses.

Moreover, the proportion of pregnant women who utilized ANC services under each of the three outcome scenarios was estimated for all of India’s states and union territories. These proportions were then compared with NITI Aayog health Index scores. The State Health Index is an annual tool to assess the performance of states and UTs. It is a weighted composite index based on 24 indicators such as Immunization coverage, ANC visits in the first trimester, Under-five mortality rates, etc., grouped under the domains of ‘Health Outcomes’, ‘Governance and Information’, and ‘Key Inputs/Processes’. Each domain has been assigned weights based on its importance with the higher score for outcome indicators [21]. Analysis was performed on STATA v15.1 (StataCorp LLC, College Station, TX, USA).

### Ethics statement

This study is the secondary data analysis of publicly available NFHS-5 data. The survey’s participants voluntarily and knowingly gave their consent. The International Institute of Population Sciences’ ethical review board granted the survey its ethical approval (IIPS). After reviewing the submitted proposal, DHS (Demographic Health Survey) granted access to the dataset.

## Results

A total of 176,877 women had a birth in the five years preceding the survey. The median (IQR) number of ANC visits attended by a woman during her previous pregnancy was 4 (IQR 3–7). A majority (59.25%) of the women reported availing of ≥ 4 antenatal care (ANC) visits during their previous pregnancy. However, 6.12% (11,366) of women reported availing no ANC visits in their last pregnancy. Moreover, only 19.49% (31,370) met the WHO recommended number of a minimum 8 ANC visits for achieving a positive pregnancy experience. Additionally, only 20.28% (35,830) women availed adequate quality of ANC services irrespective of the number of ANC visits. Amongst the different components of ANC services, 87.87% (154,235) women reported- being provided with iron-folic-acid tablets during their pregnancy while 83.62% (145,310) women received one or more doses of tetanus toxoid during their pregnancy. Additionally, 31.45% (53,054) of women were administered deworming medication before birth.

In the study sample (excluding the women who attended no ANC), more than two in three (70.55%) utilized ANC services of adequate quality during their last pregnancy. However, 14.33% of women received ANC services of inadequate quality with ≥ 4 ANC visits, while 15.12% of women received ANC services of inadequate quality with < 4 ANC visits.

Table 1 reports the stratified distribution of the ANC services utilization and adequacy of its quality among women during their previous pregnancy. More than two in three (~ 70%) women belonged to the 20–30 age group and were mostly married (98%) across all three strata. Similarly, ~ 78% were working professionals, and less than 1% of women reported consuming alcohol. Across all three strata, most women availed of ANC services from qualified health professionals and received their first ANC visit within the first trimester of pregnancy. However, 15.12% of the women had received ANC services of inadequate quality while attending less than 4 ANC visits, among whom 33.27% lacked any formal education; 42% reported no media exposure, while 82% lived in rural areas.

**Table 1:** Distribution of quality of utilization of ANC services by women belonging to the reproductive age group during their last pregnancy, NFHS-5 (2019-21)

	Inadequate quality and < 4 ANC visits (n = 24,423)	Inadequate Quality and ≥ 4 ANC visits (n = 23,144)	Adequate Quality (n = 113,930)
Age (N = 161,497)			
< 20	1608 (6.79)	1351 (6.59)	7225 (6.97)
20–30	17,084 (72.04)	16,285 (72.56)	79,981 (71.45)
31–49	5731 (21.17)	5508 (20.89)	26,724 (21.59)
**Marital Status (N = 175,240)**			
Unmarried	42 (0.16)	41 (0.12)	147 (0.06)
Married	23,981 (98.57)	22,782 (98.66)	112,192 (98.87)
Widowed/Separated/ Divorced	400 (1.27)	321 (1.22)	1591 (1.07)
**Highest educational level (N = 161,497)**			
No education	7774 (33.27)	4603 (18.33)	18,613 (15.02)
Primary	3650 (14.33)	2892 (12.07)	12,962 (10.96)
Secondary	10,961 (43.30)	12,373 (52.84)	62,625 (54.14)
Higher	2038 (9.10)	3276 (16.75)	19,730 (19.88)
**Husband’s education (N = 24,569)**			
No education	834 (22.83)	512 (12.70)	1956 (10.62)
Primary	611 (16.06)	403 (11.99)	2015 (11.98)
Secondary	1872 (49.05)	2002 (55.78)	10,079 (55.46)
Higher	482 (12.05)	590 (19.54)	3267 (19.93)
**Respondents Occupation (N = 24,670)**			
Working	979 (21.73)	874 (22.66)	4383 (21.84)
Not working	2786 (78.27)	2645 (77.34)	13,003 (78.16)
**Lifestyle factors**			
**Mass media exposure frequency (N = 161,497)**			
No exposure	10,249 (42.87)	6014 (24.58)	25,970 (21.55)
Any exposure	14,174 (57.13)	17,130 (75.42)	87,960 (78.45)
**Drinks alcohol (N = 161,497)**			
Yes	364 (0.53)	302 (0.54)	1806 (0.55)
No	24,059 (99.47)	22,842 (99.46)	112,124 (99.45)
**Tobacco consumption (Smoking/Smokeless) (N = 161,497)**			
Yes	22,806 (96.35)	21,663 (96.69)	107,454 (97.03)
No	1617 (3.65)	1481 (3.31)	6476 (2.97)
**Household Characteristics**			
**Religion (N = 161,497)**			
Hindu	17,802 (80.59)	16,800 (80.27)	86,046 (79.83)
Muslim	3589 (16.41)	3628 (15.35)	15,735 (15.47)
Other	3032 (3,00)	2716 (4.38)	12,149 (4.70)
**Caste (N = 152,521)**			
SC	5676 (27.77)	4432 (22.77)	22,218 (23.33)
ST	4744 (9.11)	4408 (10.18)	21,574 (10.64)
OBC	9766 (48.18)	8702 (45.65)	43,851 (45.27)
Non-SC/ST/OBC	3104 (14.94)	4254 (21.41)	19,792 (20.76)
**Health Insurance (N = 161,497)**			
Yes	5450 (18.18)	6363 (22.92)	33,523 (25.88)
No	18,973 (81.82)	16,781 (77.08)	80,407 (74.12)
**Type of Residence (N = 161,497)**			
Urban	3451 (17.90)	5247 (29.99)	26,698 (30.69)
Rural	20,972 (82.10)	17,897 (70.01)	87,232 (69.31)
**Region (N = 161,497)**			
North	3761 (12.20)	5407 (15.54)	22,016 (14.71)
South	1328 (6.48)	3274 (17.95)	17,239 (20.02)
Central	7820 (36.69)	5368 (25.45)	27,796 (25.48)
East	6699 (35.00)	3074 (18.36)	19,791 (23.49)
West	1074 (6.27)	3039 (19.57)	10,625 (12.99)
Northeast	3741 (3.36)	2982 (3.13)	16,463 (4.31)
**Wealth index (N = 161,497)**			
Poorest	9180 (36.32)	5118 (19.46)	24,377 (18.56)
Poor	6314 (24.71)	5277 (20.81)	25,214 (20.19)
Middle	4203 (17.59)	4920 (21.33)	23,164 (20.26)
Richer	2941 (13.11)	4391 (20.81)	21,924 (20.99)
Richest	1785 (8.28)	3438 (17.59)	19,251 (19.99)
**Pregnancy intended (N = 161,497)**			
Yes	21,705 (88.08)	21,166 (90.85)	107,183 (93.73)
No	2718 (11.92)	1978 (9.15)	6747 (6.28)
**Parity (N = 161,497)**			
1	5869 (23.59)	6715 (30.28)	43,422 (38.77)
> 2	18,554 (76.41)	16,429 (69.72)	70,508 (61.23)
**Registration of pregnancy (N = 161,497)**			
Yes	22,345 (90.93)	22,104 (94.71)	110,645 (96.77)
No	2078 (9.07)	1040 (5.29)	3285 (3.23)
**Timing of first ANC (N = 161,278)**			
First trimester	14,865 (60.09)	17,772 (77.02)	88,595 (77.42)
Second trimester	7680 (31.86)	4307 (18.11)	20,690 (17.97)
Third trimester onwards	1794 (8.05)	1014 (4.88)	4561 (4.60)
**Health Facility generally visited (N = 86,472)**			
Public	7795 (63.29)	8900 (65.98)	46,408 (68.00)
Private	3151 (35.56)	3274 (32.95)	16,342 (31.32)
Other	102 (1.15)	109 (1.07)	391 (0.68)
**ANC Provider (N = 161,313)**			
Doctor	5836 (23.14)	7002 (30.42)	31,954 (28.85)
Other qualified health professionals*	10,316 (40.96)	8951 (37.73)	44,654 (38.38)
Frontline Health worker**	8226 (35.89)	7139 (31.85)	37,235 (32.78)
**Received health checks during pregnancy (N = 118,644)**			
Yes	13,153 (84.97)	13,967 (89.75)	78,138 (91.40)
No	2618 (15.03)	1903 (10.25)	8865 (8.60)
**Told about Pregnancy complications at ANC (N = 161,497)**			
Yes	15,452 (61.85)	17,653 (74.20)	92,900 (80.79)
No	8971 (38.15)	5491 (25.80)	21,030 (19.21)
**Received counselling regarding health and nutrition (N = 99,121)**			
Yes	8206 (67.05)	9756 (73.27)	56,717 (77.72)
No	3990 (32.95)	3704 (26.73)	16,748 (22.28)
**Distance to health facility**			
No Problem	7473 (33.55)	8672 (40.08)	45,509 (43.33)
Big Problem	8064 (29.36)	6326 (24.46)	28,666 (22.54)
Not a Big Problem	8886 (37.10)	8146 (35.46)	39,755 (34.13)

Footnote:

*- Auxiliary Nurse and Midwife (ANM)/nurse/midwife/Lady Health Visitors (LHV)/CS health professional/dai/traditional birth attendant

**- community/village health worker/AWW Anganwadi worker (Integrated child development services (ICDS) worker)/ Accredited Social Health Activist (ASHA)

All percentages are weighted. All sample sizes are unweighted

The state-wise quality of ANC service utilization and its correlation with NITI Ayog Health Index scores is represented in Table 2. A significantly moderately negative correlation (r=-0.51) was found between the Niti Ayog health index scores and the inadequate-quality and less than 4 ANC visits group. However, a medium correlation (r = 0.32) was found between adequate quality ANC service utilization with the state health index score.

**Table 2:** State-wise comparative distribution of quality of ANC services utilization with NITI Ayog State Health Index

State	Health Index Reference Year (2019–2020)	Inadequate quality and < 4 ANC visits	Inadequate Quality and ≥ 4 ANC visits	Adequate Quality
High Health Index				
Kerala	82.20	36 (1.78)	126 (6.06)	1928 (92.15)
Mizoram	75.77	229 (12.85)	315 (26.24)	924 (60.91)
Tamil Nadu	72.42	91 (1.90)	763 (16.01)	4124 (82.10)
Tripura	70.16	97 (5.45)	178 (11.04)	1226 (83.50)
Telangana	69.96	420 (7.97)	827 (15.80)	4067 (76.24)
Andhra Pradesh	69.95	146 (7.17)	257 (12.27)	1651 (80.56)
Maharashtra	69.14	585 (7.82)	1474 (22.42)	4712 (69.76)
Dadra & Nagar Haveli & Daman & Diu	66.21	13 (2.11)	145 (26.89)	452 (71.00)
Gujarat	63.59	475 (6.65)	1377 (19.77)	5193 (73.57)
Himachal Pradesh	63.17	131 (6.73)	351 (17.65)	1449 (75.62)
Chandigarh	62.53	10 (6.84)	15 (9.88)	118 (83.28)
Punjab	58.08	458 (11.35)	540 (14.16)	3069 (74.48)
Karnataka	57.93	612 (8.93)	1144 (17.16)	4459 (73.91)
Sikkim	55.53	32 (7.14)	46 (9.02)	430 (83.83)
**Medium Health Index**				
Goa	53.68	1 (0.17)	43 (15.08)	268 (84.76)
Lakshadweep	51.87	0 (0.00)	9 (3.78)	232 (96.22)
Puducherry	50.83	13 (1.99)	78 (16.60)	504 (81.40)
Chhattisgarh	50.71	650 (10.51)	845 (13.04)	4631 (76.45)
Delhi	49.84	114 (5.57)	301 (14.18)	1771 (80.25)
Haryana	49.26	685 (13.08)	728 (14.41)	3505 (72.51)
Assam	47.74	1038 (12.54)	810 (9.48)	6829 (77.98)
Jharkhand	47.55	1667 (24.90)	727 (11.12)	4419 (63.99)
Jammu and Kashmir	46.99	330 (6.84)	1497 (29.17)	3180 (63.99)
**Low Health Index**				
Andaman & Nicobar islands	44.74	10 (2.07)	70 (15.41)	274 (82.52)
Odisha	44.31	291 (4.59)	639 (9.38)	6026 (86.03)
Uttarakhand	44.21	5860 (24.24)	3305 (14.09)	14,702 (61.67)
Meghalaya	43.05	822 (19.70)	883 (23.67)	2093 (56.64)
Rajasthan	41.33	1744 (17.49)	1668 (16.09)	6740 (66.42)
Madhya Pradesh	36.72	1310 (11.86)	1218 (10.71)	8463 (77.43)
Manipur	34.26	216 (5.77)	247 (11.30)	1776 (82.93)
Arunachal Pradesh	33.92	803 (22.80)	407 (11.18)	2320 (66.02)
Bihar	31.00	4567 (40.61)	1267 (11.34)	5545 (48.06)
Uttar Pradesh	30.57	289 (10.61)	307 (13.13)	2184 (76.27)
Nagaland	27.01	504 (32.06)	96 (8.34)	865 (59.60)
West Bengal	Not Available	174 (3.63)	441 (10.06)	3810 (86.31)

On bivariate analysis, the following variables were significantly associated with the quality of utilization of ANC services: marital status, women’s education levels, exposure to mass media, caste, religion, health insurance, wealth index, type of residence, and region. Other maternal factors such as pregnancy intention, parity, ANC provider and distance to health facility also significantly affected the likelihood of utilizing ANC services of adequate quality (Table 3).

**Table 3:** Multinominal regression analysis of factors associated with utilization of ANC services and adequacy of ANC quality among women in their previous pregnancy in India, NFHS 5 (2019-21) (N = 152,347)

Factors	Inadequate quality and < 4 ANC visits*		Inadequate Quality and ≥ 4 ANC visits*	
	RRR (95% CI)	aRRR (95% CI)	RRR (95% CI)	aRRR (95% CI)
Age				
< 20	1	1	1	1
20–30	1.03(0.96,1.11)	0.88 (0.81,0.96)	1.07 (0.98,1.17)	0.87 (0.79,0.95)
31–49	1.00 (0.92,1.09)	0.73 (0.66,0.80)	1.02 (0.93,1.12)	0.75 (0.67,0.82)
**Marital Status**				
Unmarried	1	1	1	1
Married	0.39 (0.22,0.67)	0.33 (0.16,0.67)	0.54 (0.31,0.94)	0.54 (0.30,0.96)
Widowed/Separated/ Divorced	0.46 (0.25,0.83)	0.40 (0.19,0.85)	0.62 (0.34,1.11)	0.61 (0.33,1.13)
**Highest educational level**				
No education	1	1	1	1
Primary	0.59 (0.56,0.63)	0.73 (0.69, 0.78)	0.90 (0.94,0.97)	0.93 (0.86,1.00)
Secondary	0.36 (0.34,0.38)	0.61 (0.58,0.65)	0.80 (0.76,0.84)	0.87 (0.81,0.92)
Higher	0.21 (0.19,0.22)	0.54 (0.49,0.59)	0.69 (0.64,0.74)	0.86 (0.79,0.93)
**Lifestyle factors**				
**Mass media exposure frequency**				
No exposure	1	1	1	1
Any exposure	0.37 (0.35,0.38)	0.69 (0.66,0.73)	0.84 (0.80,0.88)	0.88 (0.84,0.94)
**Household Characteristics**				
**Religion**				
Hindu	1	1	1	1
Muslim	1.05 (0.98,1.13)	1.02 (0.95,1.11)	0.99 (0.92,1.05)	0.99 (0.92,1.06)
Other	0.63 (0.57,0.70)	1.00 (0.89,1.11)	0.93 (0.84,1.02)	0.98 (0.87,1.08)
**Caste**				
SC	1.65 (1.53,1.78)	1.22 (1.13,1.31)	0.95 (0.88,1.02)	0.92 (0.85,0.98)
ST	1.19 (1.09,1.30)	0.79 (0.72,0.87)	0.93 (0.85,1.01)	0.83 (0.76,0.91)
OBC	1.48 (1.38,1.58)	1.29 (1.21,1.38)	0.98 (0.92,1.04)	0.98 (0.92,1.05)
Non-SC/ST/OBC	1	1	1	1
**Health Insurance**				
Yes	0.64 (0.60,0.67)	0.71 (0.68,0.75)	0.85 (0.81,0.89)	0.83 (0.79,0.87)
No	1	1	1	1
**Type of Residence**				
Urban	1	1	1	1
Rural	2.03 (1.88,2.19)	1.19 (1.09,1.30)	1.03 (0.97,1.10)	0.98 (0.92,1.05)
**Region**				
North	1	1	1	1
Central	1.62 (1.50,1.74)	1.12 (1.03,1.20)	0.88 (0.83,0.94)	0.76 (0.71,0.82)
East	1.67 (1.54,1.82)	1.09 (1.01,1.19)	0.69 (0.64,0.74)	0.58 (0.54,0.63)
Northeast	0.88 (0.79,0.97)	0.61 (0.54,0.68)	0.64 (0.59,0.70)	0.66 (0.60,0.74)
West	0.54 (0.48,0.61)	0.49 (0.43,0.55)	1.32 (1.22,1.44)	1.23 (1.13,1.34)
South	0.36 (0.33,0.40)	0.35 (0.31,0.39)	0.79 (0.74,0.85)	0.79 (0.73,0.85)
**Wealth index**				
Poorest	1	1	1	1
Poorer	0.63 (0.59,0.66)	0.84 (0.79,0.89)	0.98 (0.93,1.04)	0.97 (0.91,1.03)
Middle	0.44 (0.42,0.47)	0.79 (0.74,0.84)	1.00 (0.94,1.07)	0.97 (0.90,1.05)
Richer	0.32 (0.30,0.34)	0.68 (0.63,0.74)	0.95 (0.89,1.01)	0.89 (0.82,0.97)
Richest	0.21 (0.19,0.23)	0.52 (0.47,0.58)	0.84 (0.78,0.90)	0.81 (0.73,0.90)
**Pregnancy intended**				
Yes	0.50 (0.46,0.53)	0.62 (0.58,0.66)	0.67 (0.62,0.72)	0.70 (0.65,0.75)
No	1	1	1	1
**Parity**				
1	1	1	1	1
> 2	2.05 (1.97,2.14)	1.69 (1.60,1.78)	1.46(1.40,1.52)	1.43 (1.36,1.51)
**ANC Provider**				
Doctor	1	1	1	1
Other qualified health professionals^#^	1.33 (1.26,1.41)	0.93 (0.87,0.98)	0.93 (0.87,0.99)	0.93 (0.87,0.99)
Frontline Health worker^##^	1.36 (1.28,1.45)	0.73 (0.69,0.77)	0.92 (0.87,0.98)	0.92 (0.86,0.98)
**Distance to health facility**				
No problem	1	1	1	1
Big problem	1.68 (1.59,1.78)	1.14 (1.07,1.21)	1.17 (1.11,1.24)	1.13 (1.07,1.20)
Not a big problem	1.40 (1.34,1.48)	1.11 (1.05,1.17)	1.12 (1.07,1.18)	1.10 (1.04,1.16)

Footnote:

aRRR: adjusted relative risk ratio; adjusted for all variables from bivariate analysis

^#^- Auxiliary Nurse and Midwife (ANM)/nurse/midwife/Lady Health Visitors (LHV)/CS health professional/dai/traditional birth attendant

^##^- community/village health worker/AWW Anganwadi worker (Integrated child development services (ICDS) worker)/ Accredited Social Health Activist (ASHA)

*With reference to the utilization of ANC services of adequate quality

On adjusted analysis, women aged between 31 and 49 years were significantly less likely (aRRR 0.73 95% CI 0.66, 0.80) to utilize ANC services of inadequate quality, and < 4 ANC visits. Additionally, any exposure to mass media (aRRR 0.69 95% CI 0.66, 0.73), and having health insurance (aRRR 0.71 95% CI 0.68, 0.75) decreased their risk of receiving ANC services of inadequate quality and < 4 ANC visits. Women belonging to the richest wealth quintile (aRRR 0.52 95% CI 0.47,0.58) were at significantly lower risk of utilizing ANC services of inadequate quality and < 4 ANC visits. However, multiparous women (aRRR 1.69 95% CI 1.60, 1.78) had significantly higher likelihood of utilizing ANC services of inadequate quality and obtaining < 4 ANC visits, compared to the primigravida. Similarly, women with an intended pregnancy had significantly lower risk (aRRR 0.62 95% CI 0.58,0.66) of receiving ANC services of inadequate quality and obtaining < 4 ANC visits. Women’s residence in eastern India (aRRR 1.09 95% CI 1.01, 1.19) was associated with higher risk of receiving ANC services of inadequate quality and obtaining < 4 ANC visits. Finally, the mothers who reported distance to health facility as a “big problem” were at significantly higher likelihood of utilizing ANC services of inadequate quality irrespective of their number of ANC visits.

Similarly, on adjusted analysis, women who had any health insurance (aRRR 0.83 95% CI 0.79,0.87) and belonged to richest wealth quintile (aRRR 0.81 95% CI 0.73,0.90) were less likely to utilize inadequate quality ANC services and attend ≥ 4 ANC visits. Additionally, compared to women in < 20 age group, women belonging to 20–30 age group (aRRR 0.87 95% CI 0.79,0.95) or 31–49 age group (aRRR 0.75 95% CI 0.67,0.82) were less likely to utilize inadequate quality ANC services and attend ≥ 4 ANC visits. Furthermore, women who reported any media exposure (aRRR 0.88 95% CI 0.84,0.94) had significantly lower likelihood of utilizing inadequate quality of ANC services. However, women who were multiparous (aRRR 1.43 95% CI 1.36,1.51) or lived in western India (aRRR 1.23 95% CI 1.13,1.34) or for mothers who had a big problem with distance to health facility (aRRR 1.13 95% CI 1.07,1.20) were significantly more likely of utilizing inadequate quality of ANC services (Table 3).

**Fig. 2:**
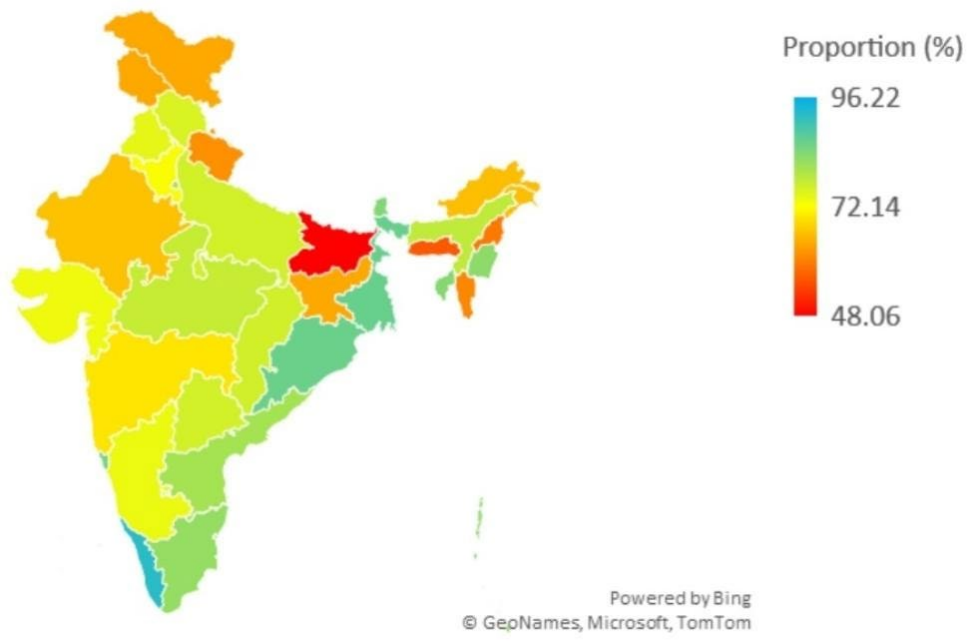
Statewise distribution of utilization of adequate quality ANC services, NFHS-5,2019-21

The state-wise distribution of adequate ANC quality service utilization has been depicted in Fig. 2. A higher proportion of utilization of adequate quality service is observed in Kerala, Goa, Orissa and West Bengal. Lower proportion of adequate quality utilization is observed in Bihar and the northeastern states such as Meghalaya, Nagaland.

## Discussion

Despite various programmatic interventions by the government of India to promote maternal health, the study findings indicate that only three in five women complete the conservatively recommended number of ≥ 4 ANC visits during pregnancy, while only one in five women availed adequate quality of ANC services. However, the ANC coverage in India has significantly improved from 51.6% (NFHS-4) to 59.25% (NFHS-5). The coverage is similar to that observed in other lower middle-income countries like Ghana (58.61%) [22] but is lower than the global average of 61.8% [23]. However, only one in five women in NFHS-5 completed the WHO positive pregnancy recommended guideline for attending a minimum of 8 ANC visits during pregnancy. Furthermore, there were ~ 6% women who reported attending no ANC visits during their previous pregnancy indicative of a high-risk group that warrants focused policy intervention.

Higher education of women and older age at pregnancy significantly decreased the likelihood of women utilizing inadequate quality ANC services. These findings are in line with studies in other neighboring countries of India like Nepal [18] and Bangladesh [24]. Another study in Malaysia also observed that women with no education or primary education were less likely to have adequate antenatal care utilization compared to women with tertiary education [25, 26].

Education enables mothers with the necessary knowledge and sensitization regarding the importance of ANC services and the negative impact associated with their non-utilization while improving agency and empowerment to make their own decisions regarding healthcare utilization during pregnancy [12].

Living in urban areas and belonging to richer wealth quintiles significantly increases the likelihood of availing adequate quality ANC services, a finding in agreement with existing literature from India [12] and Malawi [25]. Moreover, in LMICs like India, women belonging to higher wealth quintile have higher financial and social capital which improves the care utilization patterns [23]. Living in urban areas and possessing more wealth may accentuate other enablers of optimal quality ANC service due to its correlation with better educational background and having higher access to mass media leading to greater awareness in women of the beneficial effects of ANC services in improving pregnancy outcomes [27]. Consequently, this signifies the need for strengthening ANC health promotion campaigns in rural and remote regions of the country.

Based on our data, there is a notable disparity in the utilization of antenatal care (ANC) services of adequate quality across different regions within the country. Women living in Northern India were less likely to utilize ANC services of adequate quality compared to women living in Southern India. This study also corroborates previous evidence documenting highest utilization of ANC services in states of south India like Kerala and Tamil Nadu having higher human development indices [28, 29].

In this study, multiparous women reported more likelihood of availing inadequate quality ANC services. This could be due to financial constraints imposed by multiple children or could be due to prior experience with childbirth contributing to complacency which drives the erroneous perception of limited benefit of regular ANC visits during pregnancy. Previous studies in India and other LMICS have also observed this phenomenon suggestive of the need for reinvigorating ANC campaigns with key messages emphasizing their necessity for birth preparedness and complication readiness [12, 30].

Our study findings emphasise the importance of quality of ANC services utilised irrespective of number of ANC visits availed. For instance, in our study women with higher educational levels were less likely to avail inadequate quality of ANC services irrespective of number of ANC visits. In line with our findings, a recent mutlinational study also revealed that frequent ANC visits of inadequate quality do not suffice the objective of reducing maternal and infant mortality [31].

Our study findings observed a moderate correlation between NITI Ayog Health Index and the proportion of adequate quality of ANC utilization. Although the NITI Ayog Health Index incorporates primarily multiple MCH indicators, it does not focus on the quality or content of services being provided. Therefore, a comprehensive indicator that not only considers the number of visits but also incorporates a measure of the quality of ANC services being delivered at the facility by means of quantitative indicators is recommended [32].

Despite increase in ANC coverage in LMICs globally, a disproportionately higher burden of perinatal and maternal deaths has been reported even among women who utilize the minimum requirement of 4 ANC visits [1], The World Health Organization in 2016, recommended a new ANC model which increases the minimum number of ANC contacts during pregnancy to 8 visits: five contacts in the third trimester, one contact in the first trimester, and two contacts in the second trimester. However, our analysis indicated that close to 40% of women in India are still unable to attend at least 4 ANC visits. Considering this, the aim of achieving a further rapid reduction in maternal mortality will be extremely challenging in settings with existing low ANC utilization [33].

Over the years, the government of India (both central and state governments) have launched various health programs including the landmark National Health Mission, Janani Shishu Suraksha Karyakram, Janani Suraksha Yojana, Pradhan Mantri Surakshit Matritva Abhiyan with the goals of improving ANC service accessibility, coverage, minimize out of pocket costs during pregnancy, early registration of pregnancy to enable birth preparedness and complication readiness, and promote institutional delivery even in resource limited settings through strengthening of first referral units. However, evidence from this study highlights the likely gap in service delivery and implementation of these programs apart from the persistent regional disparity in utilization of ANC services across the country.

Consequently, the present study has the following recommendations for the Reproductive and Maternal health component of India’s RMNCH programme. First, women in India from socioeconomically disadvantaged groups are substantially less likely to access and utilize ANC services, especially that of adequate quality. Regions in India with higher proportion of women from low SES groups therefore require special focus for strengthening antenatal care related activities. Second, women with exposure to mass media are more likely to utilize ANC services, suggestive of the need for greater and effective utilization of the medium to improve individual awareness and community sensitization of the pressing need for early initiation and regular ANC visits to improve pregnancy related outcomes. For women with no education or no exposure to mass media, targeted health education campaigns to improve understanding of the need for utilization of ANC services is crucial. Third, women with unintended pregnancies are less likely to utilize ANC visits of adequate quality. Frontline health workers therefore have a pivotal role in ensuring that these women have early contact with the health system for promoting up-take of ANC services. Fourth, a substantial proportion of women in India despite attending 4 ANC visits fail to attain ANC of adequate quality suggestive of the need for health system strengthening activities that promote regular and prompt delivery of ANC services. Additionally, improving the regular monitoring and evaluation of existing maternal and child health programs is needed through specialized training and supervision of health care providers. Furthermore, the facilities covered under government health insurance schemes, especially the Ayushman Bharat National Health Protection Scheme should consider incorporating high-quality ANC services. Programmatic initiatives for the deployment of group ANC services should also be explored in Indian settings [34].

The major strength of this study is the large, nationally representative study sample that was collected through trained field investigators. However, there exist certain study limitations. Important parameters to assess the quality of ANC service including Level-II Ultrasound services, management of severe anemia and complications during pregnancy, and the administration of prophylactic antimalarial medicines (IPTp) in high burden areas were not available. Additionally, the conceptual framework used for health service utilization required an assessment of the perceived need for ANC service utilization among women. Moreover, respondents might have given socially desirable answers to questions that measured behavioral aspects of their health during their last pregnancy. Additionally, as most of the data points are patient-reported, study findings can be subject to a significant recall bias.

## Conclusion

Although nearly 3 in 5 women in India utilized a minimum mandated ≥ 4 ANC visits during their last pregnancy, only one in five of those received adequate quality of ANC services indicating suboptimal content. Furthermore, only one in five women utilized the WHO mandated ≥ 8 ANC visits for a positive pregnancy experience. Furthermore, 14.3% of the women received ANC services of inadequate quality despite attending ≥ 4 ANC visits in their previous pregnancy. Our study emphasized the importance of quality of ANC services utilised irrespective of number of ANC visits availed. Efforts should be undertaken to enhance the utilization of antenatal care (ANC) services by implementing media initiatives that aim to raise awareness, particularly among women belonging to disadvantaged population groups. Furthermore, stringent monitoring and evaluation of existing maternal and child health programs is needed to improve quality of ANC service utilization.

### Electronic supplementary material

Below is the link to the electronic supplementary material.

Supplementary Material 1

## Data Availability

All data used in this analysis can be downloaded directly from the DHS program (after registration) at: https://dhsprogram.com/data/available-datasets.cfm.
