# Supplementary material for: Utilization and determinants of adequate quality antenatal care services in India: evidence from the National Family Health Survey (NFHS-5) (2019-21)
Source: BMC Pregnancy Childbirth. 2023 Nov 17;23:800. doi: 10.1186/s12884-023-06117-z (PMC10657001; doi:10.1186/s12884-023-06117-z)
Supplement: Supplementary file 1 — Supplementary Material 1 [file 12884_2023_6117_MOESM1_ESM.docx]

**Appendix I**

**Mass media exposure:** Categorized as ‘Yes’ for females who either read newspapers or magazines listen to the radio or watch television compared to those who did not fall into any of the above scenarios.

Independent variables with several categories were clubbed into meaningful alternatives:

**Marital status**: It was integrated into three categories:- unmarried, married, divorced/separated/widowed. The occupation was categorized as working or non-working. The place of delivery was categorized as public, private, and at home.

**ANC Providers** were categorized as doctors, Other qualified healthcare professionals (Nurses, ANM, midwives, LHV, CS health professional, dai, traditional birth attendant), and Frontline health workers (Community, village health worker, Anganwadi, Integrated child development services (ICDS) worker, ASHA). ICDS is a supplementary nutritional program which caters to pregnant and lactating women.

**Caste:** The community was classified as Scheduled Caste (SC), Scheduled Tribe, Other Backward Castes (OBCs), and others. The SC and ST communities are officially considered as representing historically, the most socio-economically disadvantaged groups in India.

**Region:** The region was classified based on the state variable as follows:

- North: Jammu & Kashmir, Himachal Pradesh, Punjab, Chandigarh, Uttarakhand, Haryana, NCT of Delhi, Rajasthan, and Ladakh.
- Central: Uttar Pradesh, Madhya Pradesh, Chhattisgarh.
- East: Bihar, West Bengal, Jharkhand, and Orissa.
- Northeast: Sikkim, Arunachal Pradesh, Nagaland, Manipur, Mizoram, Tripura, Meghalaya, and Assam.
- West: Gujarat, Dadra & Nagar haveli, daman & diu, Maharashtra, and Goa.
- South: Andhra Pradesh, Karnataka, Lakshadweep, Kerala, Tamil Nadu, Puducherry, Andaman & Nicobar Islands, Telangana.
